# Supplementary figures and images for: Single nucleus RNA sequencing of the cerebral cortex from genetically diverse inbred mouse strains reveals differences in pericyte and endothelial cell composition
Source: PLoS One. 2025 Aug 28;20(8):e0323827. doi: 10.1371/journal.pone.0323827 (PMC12393717; doi:10.1371/journal.pone.0323827)

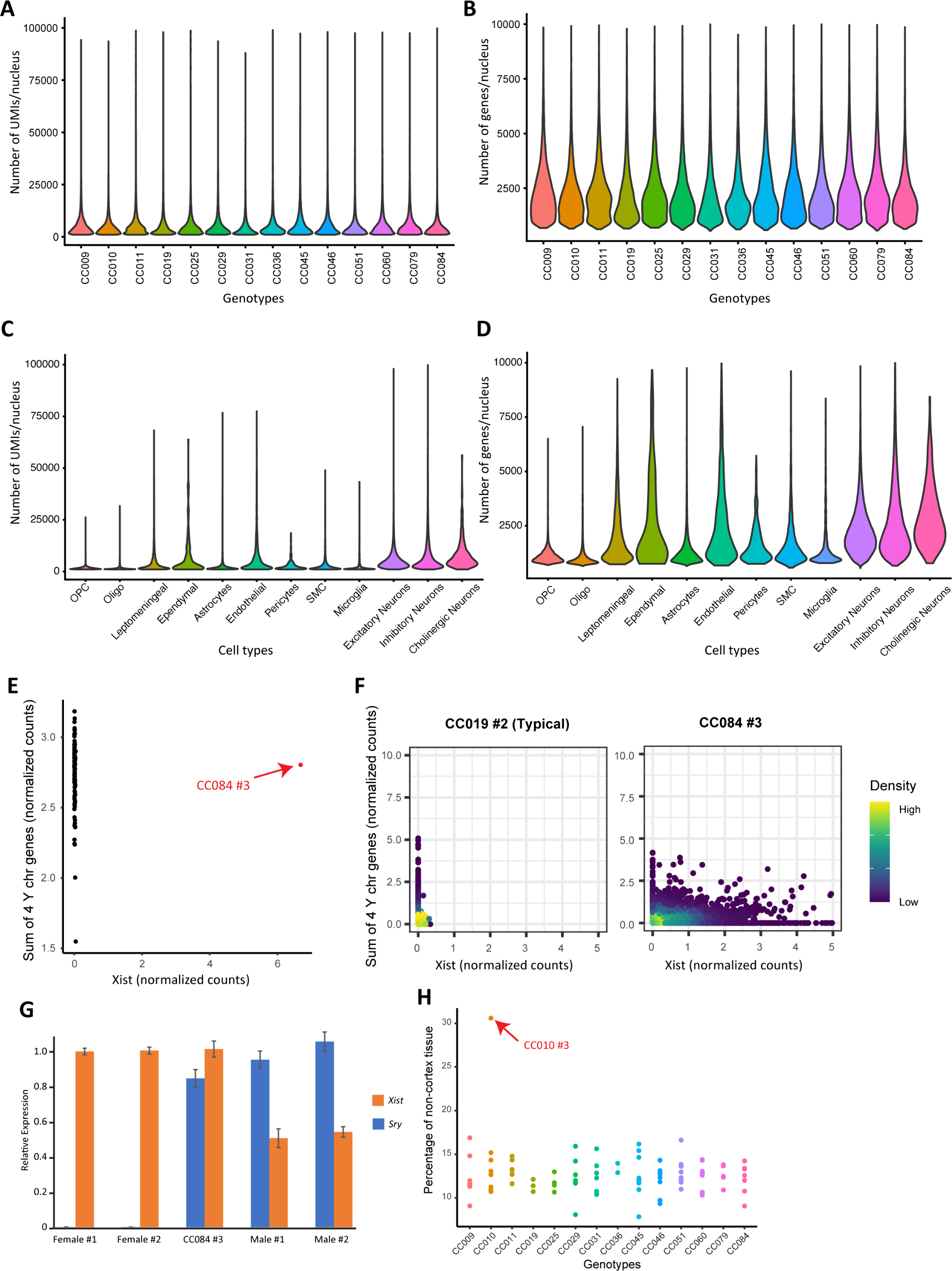

Supplement: S1 Fig — (A) Violin plot showing number of UMIs per nucleus across each CC strain. (B) Violin plot showing number of genes per nucleus across each CC strain. (C) Violin plot showing number of UMIs per nucleus across each principal cell type. (D) Violin plot showing number of genes per nucleus across each principal cell type. (E) Scatter plot showing normalized counts for Xist (female-specific gene on X-chromosome) and four Y-chromosome genes (Uty, Kdm5d, Ddx3y, Eif23y) for each sample. Normalized counts are calculated by dividing the total counts of the genes of interest by the total counts of all expressed genes and multiplying by 10,000. (F) 2D density scatter plot showing normalized counts for Xist and the four Y-chromosome genes (Uty, Kdm5d, Ddx3y, Eif23y) for each nucleus for typical samples (representative sample CC019 #2 is shown) and an outlier sample (CC084 #3; presumed XXY male). (G) Quantitative PCR of Sry and Xist genes in two female, two male samples and CC083 #3 outlier sample. (H) Percentage of non-cortex tissue for each sample. One outlier sample CC010 #3 with a high percentage of non-cortex tissue is marked with an arrow. (TIF) [file pone.0323827.s004.tif]

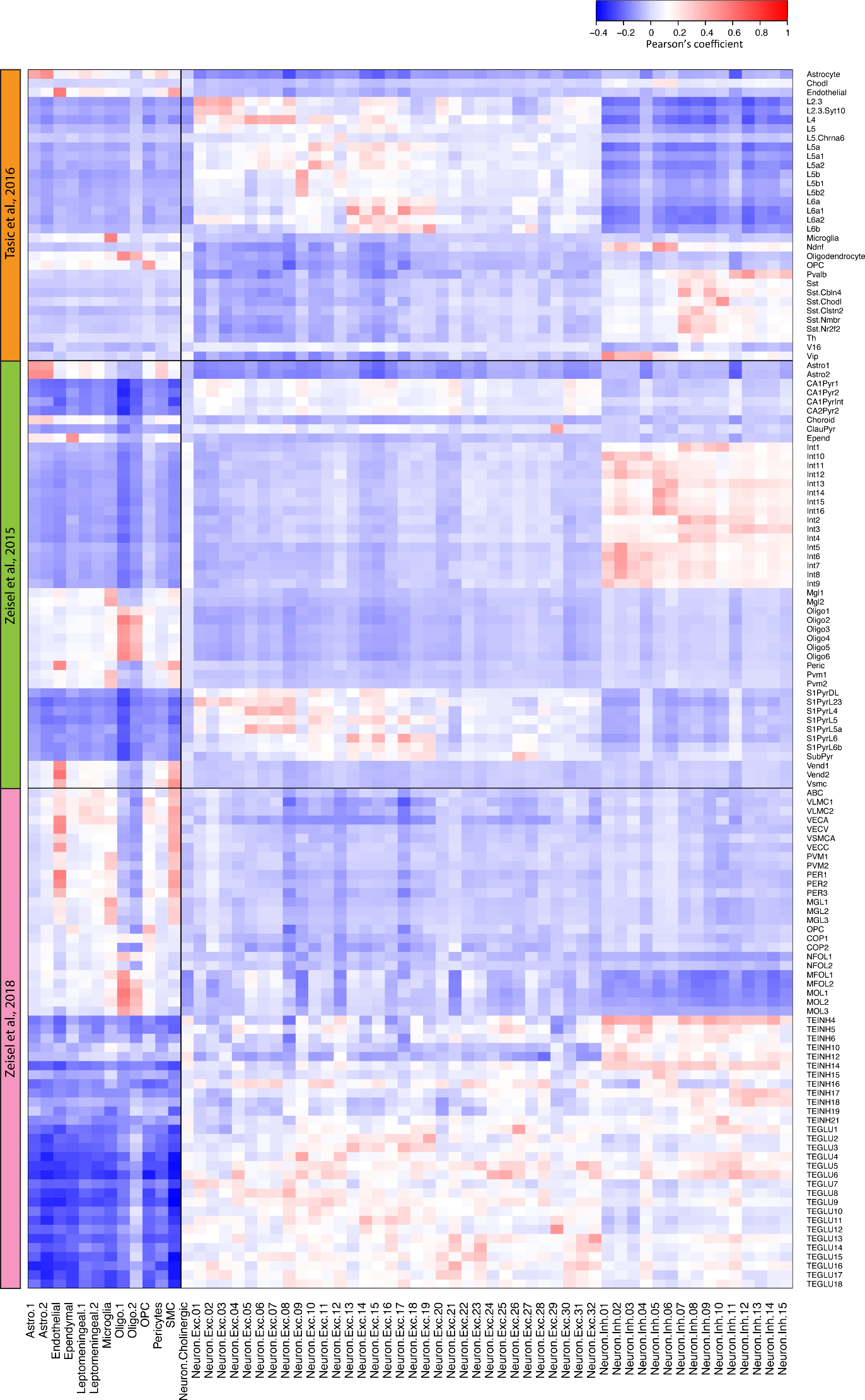

Supplement: S2 Fig — Heatmap showing Pearson correlation between the average normalized gene expression from our brain cell types and those from Tasic et al. 2016, Zeisel et al. 2015 and Zeisel et al. 2018. (TIF) [file pone.0323827.s005.tif]

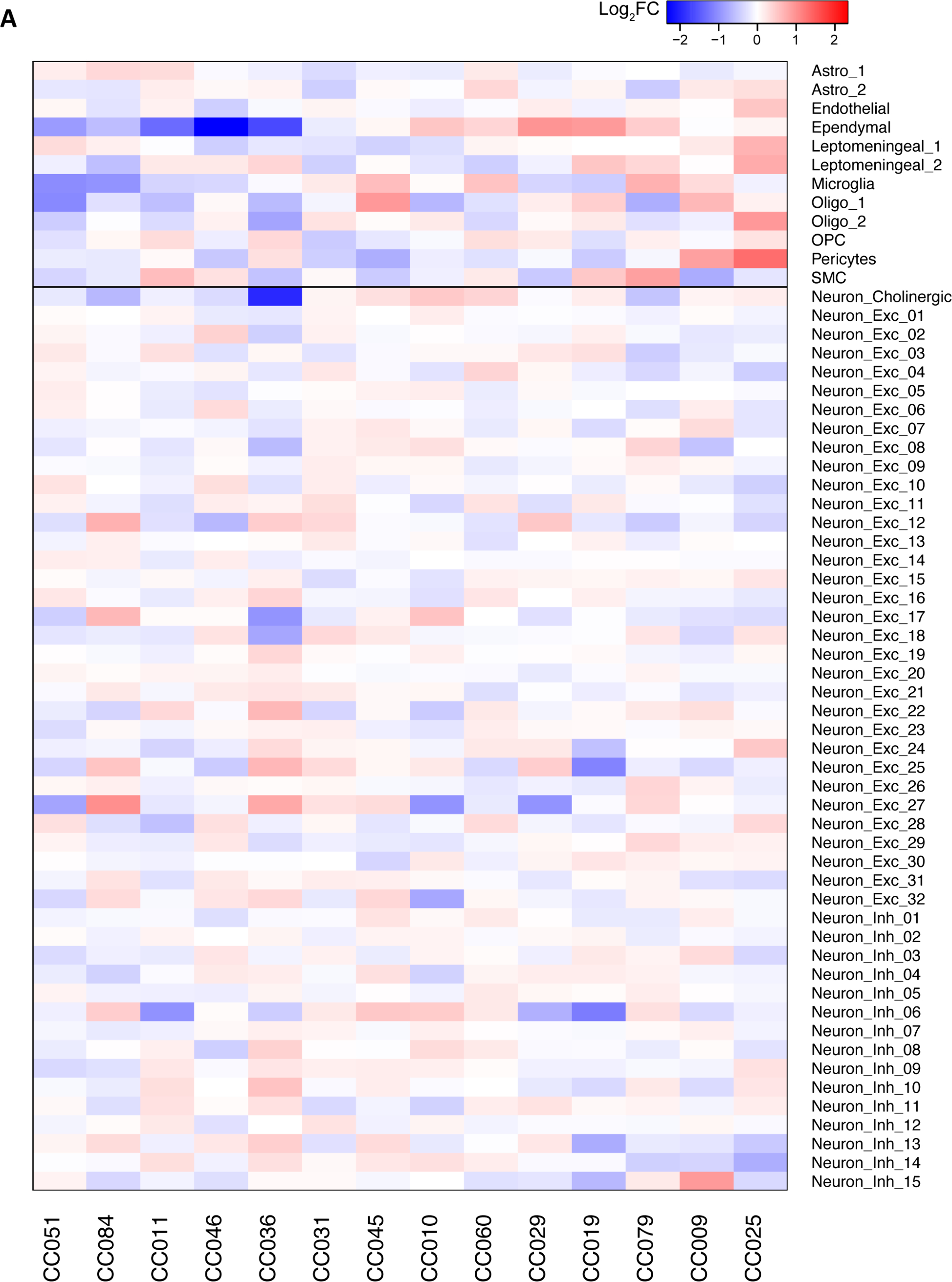

Supplement: S3 Fig — (A) Heatmap showing 60 refined cell type composition differences across the CC strains. Log2FC of each cell type for each strain was calculated by comparing to the average proportion of the corresponding cell type across all strains. (TIF) [file pone.0323827.s006.tif]

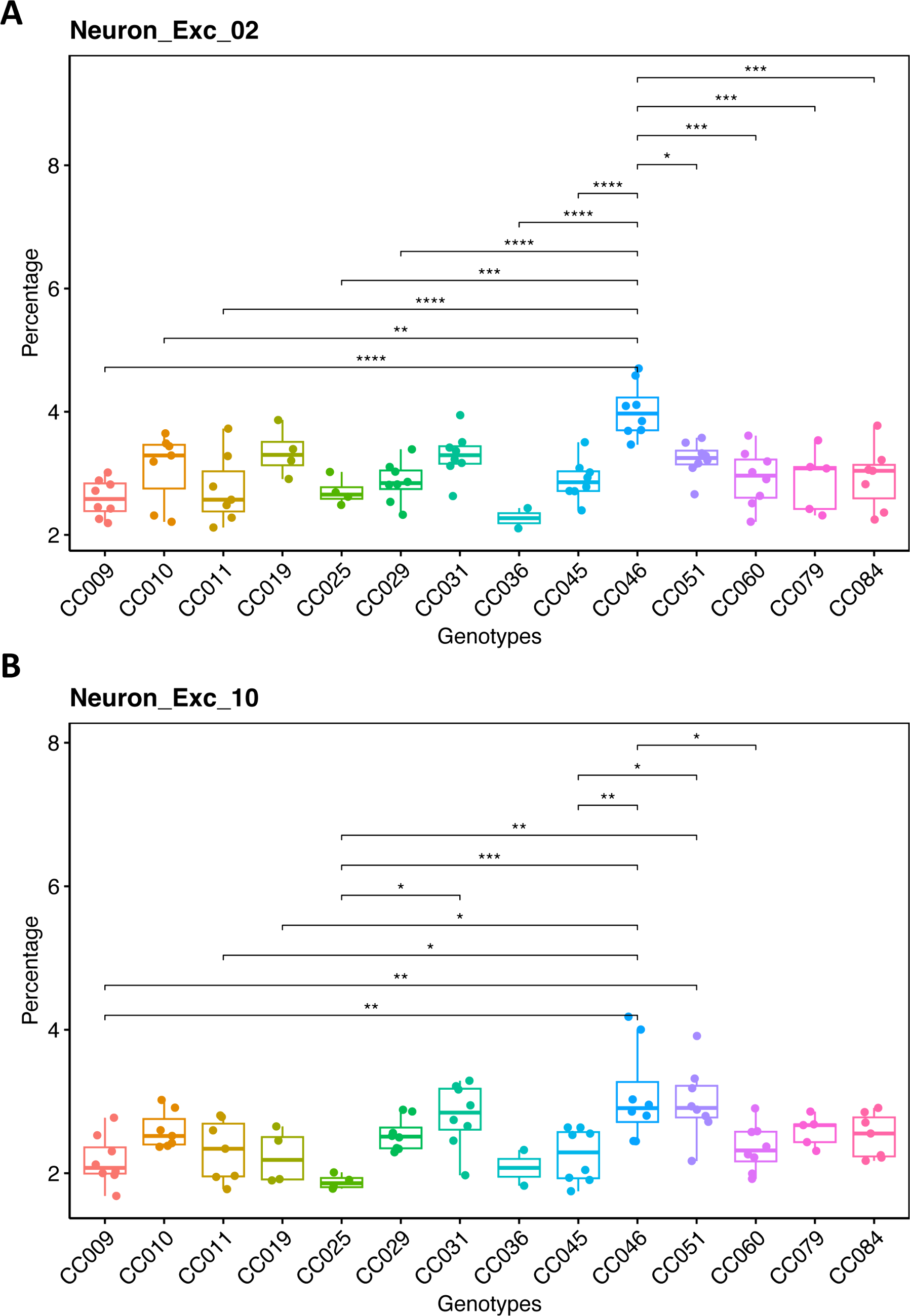

Supplement: S4 Fig — (A-B) Boxplot showing cell composition of (A) Neuron_Exc_02 and (B) Neuron_Exc_10 in each strain. Each dot represents each sample in a given strain. Statistical significance was calculated by Tukey’s HSD posthoc tests. * p < 0.05; ** p < 0.01; *** p < 0.001. (TIF) [file pone.0323827.s007.tif]

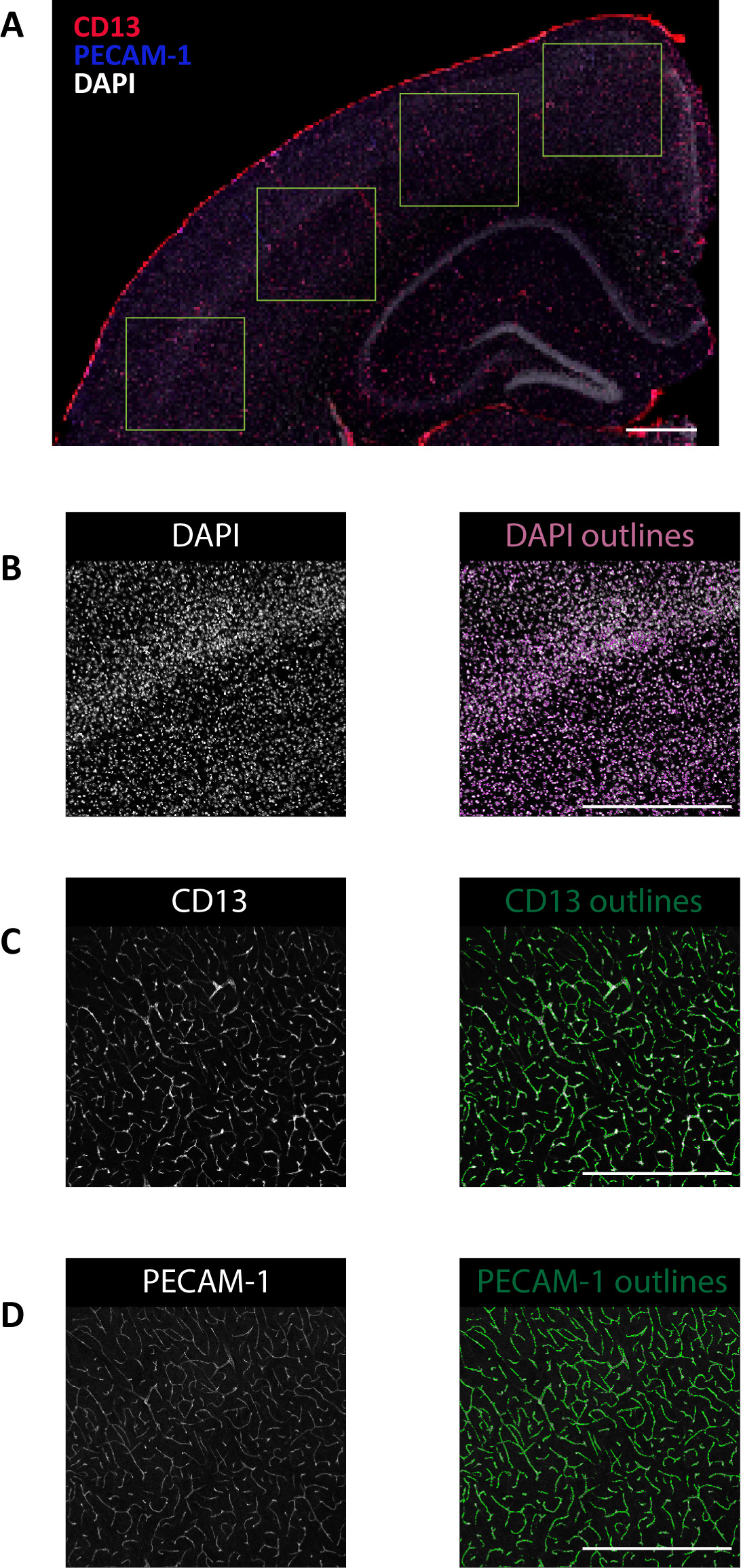

Supplement: S5 Fig — (A) Representative image of a CC brain cortex section. Green rectangles mark the regions of interest used to quantify areas positive for DAPI, CD13 and PECAM-1 staining. (B) Left panel shows DAPI signal from the immunostained brain section. Right panel shows DAPI positive area (purple) recognized by CellProfiler. (C) Left panel shows CD13 signal from the immunostained brain section. Right panel shows CD13 positive area (green) recognized by CellProfiler. (D) Left panel shows PECAM-1 signal from the immunostained brain section. Right panel shows PECAM-1 positive area (green) recognized by CellProfiler. (TIF) [file pone.0323827.s008.tif]

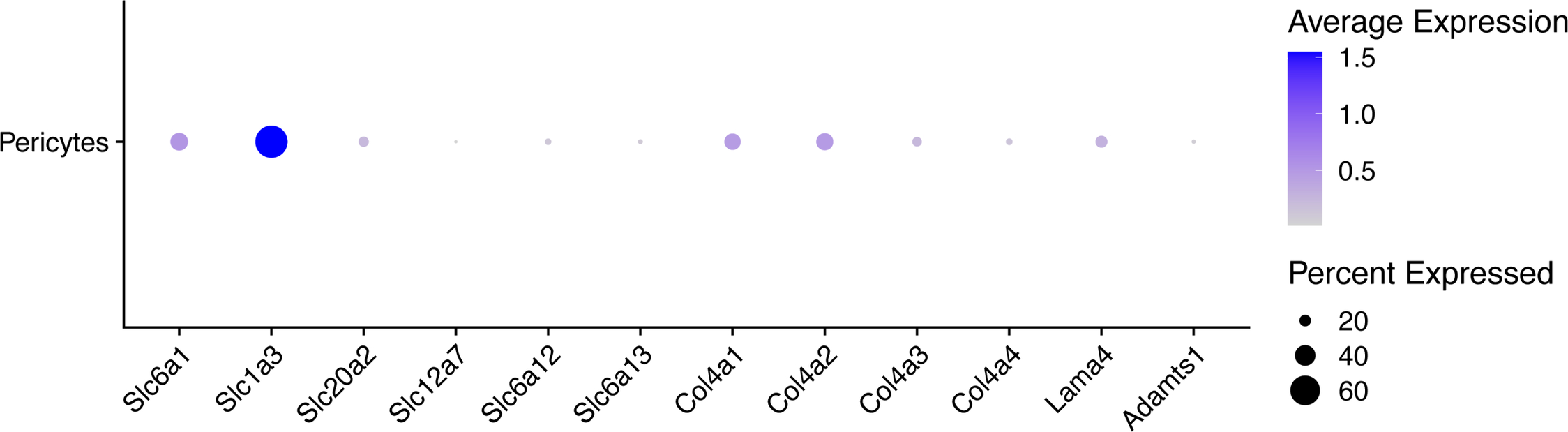

Supplement: S6 Fig — Dot Plot showing the expression of -pericyte markers (Slc6a1, Slc1a3, Slc20a2, Slc12a7, Slc6a12, Slc6a13) and M-pericyte markers (Col4a1, Col4a2, Col4a3, Col4a4, Lama4, Adamts1) within the identified pericyte cluster. (TIF) [file pone.0323827.s009.tif]
