# Supplementary material for: Single nucleus RNA sequencing of the cerebral cortex from genetically diverse inbred mouse strains reveals differences in pericyte and endothelial cell composition
Source: PLoS One. 2025 Aug 28;20(8):e0323827. doi: 10.1371/journal.pone.0323827 (PMC12393717; doi:10.1371/journal.pone.0323827)
Supplement: Attachment — Submitted filename: Response to Reviewers_final.docx [file pone.0323827.s011.docx]

**Response to Reviewers**

Journal Requirements:

When submitting your revision, we need you to address these additional requirements.

1. Please ensure that your manuscript meets PLOS ONE's style requirements, including those for file naming. The PLOS ONE style templates can be found at

<https://journals.plos.org/plosone/s/file?id=wjVg/PLOSOne_formatting_sample_main_body.pdf> and

<https://journals.plos.org/plosone/s/file?id=ba62/PLOSOne_formatting_sample_title_authors_affiliations.pdf>

- We ensured that our manuscript meets PLOS ONE’s style requirements.

2. To comply with PLOS ONE submissions requirements, in your Methods section, please provide additional information regarding the experiments involving animals and ensure you have included details on (1) methods of sacrifice, and (2) efforts to alleviate suffering.

- We have updated the Methods section to describe the procedures used to sacrifice animals and minimize suffering. Perfusion under deep anesthesia was used as the method of euthanasia, in accordance with institutional guidelines and approved protocols.

Updated original methods text: “To isolate nuclei from brain tissue, mice were deeply anesthetized with pentobarbital sodium (50 mg/kg, intraperitoneal injection). Anesthesia depth was confirmed by the absence of a response to toe-pinch. To minimize suffering, all animals were perfused transcardially with ice-cold phosphate-buffered saline (PBS) while under deep anesthesia. Perfusion served as the method of euthanasia. Following perfusion, the right cortical hemisphere was dissected and homogenized for nuclei isolation.”

3. Thank you for stating the following financial disclosure:
“J.P. is supported by grants from the National Institute of Child Health and Human Development (NICHD; T32HD040127). Microscopy was performed at the UNC Neuroscience Microscopy Core (RRID:SCR_019060), supported, in part, by funding from the NIH-NINDS (P30 NS045892) and the NIH-NICHD (P50 HD103573). M.J.Z. is supported by grants from the NINDS, NIMH, and NIEHS (R01NS109304, R01MH120229, R35ES028366).”

Please state what role the funders took in the study. If the funders had no role, please state: "The funders had no role in study design, data collection and analysis, decision to publish, or preparation of the manuscript."
If this statement is not correct you must amend it as needed.
Please include this amended Role of Funder statement in your cover letter; we will change the online submission form on your behalf.

- We have included the following statement in our cover letter to address the journal’s requirement regarding the role of funders:

“J.P. is supported by grants from the National Institute of Child Health and Human Development (NICHD; T32HD040127). Microscopy was performed at the UNC Neuroscience Microscopy Core (RRID:SCR_019060), supported, in part, by funding from the NIH-NINDS (P30 NS045892) and the NIH-NICHD (P50 HD103573). M.J.Z. is supported by grants from the NINDS, NIMH, and NIEHS (R01NS109304, R01MH120229, R35ES028366). The funders had no role in study design, data collection and analysis, decision to publish, or preparation of the manuscript.”

4. Thank you for stating the following in the Acknowledgments Section of your manuscript:
“We thank Zylka lab members for technical assistance. J.P. is supported by grants from the National Institute of Child Health and Human Development (NICHD; T32HD040127). Microscopy was performed at the UNC Neuroscience Microscopy Core (RRID: SCR_019060), supported, in part, by funding from the NIH-NINDS (P30 NS045892) and the NIH-NICHD (P50 HD103573). M.J.Z. is supported by grants from the NINDS, NIMH, and NIEHS (R01NS109304, R01MH120229, R35ES028366).”

We note that you have provided funding information that is not currently declared in your Funding Statement. However, funding information should not appear in the Acknowledgments section or other areas of your manuscript. We will only publish funding information present in the Funding Statement section of the online submission form.
Please remove any funding-related text from the manuscript and let us know how you would like to update your Funding Statement. Currently, your Funding Statement reads as follows:
“J.P. is supported by grants from the National Institute of Child Health and Human Development (NICHD; T32HD040127). Microscopy was performed at the UNC Neuroscience Microscopy Core (RRID:SCR_019060), supported, in part, by funding from the NIH-NINDS (P30 NS045892) and the NIH-NICHD (P50 HD103573). M.J.Z. is supported by grants from the NINDS, NIMH, and NIEHS (R01NS109304, R01MH120229, R35ES028366).”

Please include your amended statements within your cover letter; we will change the online submission form on your behalf.

- We have also addressed the funding placement concern. The revised Acknowledgments section, with funding details removed, reads:

“We thank Zylka lab members for technical assistance.”

All funding-related information has been retained solely in the Funding Statement section, and we confirm that the current Funding Statement contains all necessary funding acknowledgments. Therefore, no further changes to the Funding Statement are required.

5. In the online submission form, you indicated that “Additional data supporting the findings of this study are available from the corresponding author upon reasonable request.”

All PLOS journals now require all data underlying the findings described in their manuscript to be freely available to other researchers, either 1. In a public repository, 2. Within the manuscript itself, or 3. Uploaded as supplementary information.
This policy applies to all data except where public deposition would breach compliance with the protocol approved by your research ethics board. If your data cannot be made publicly available for ethical or legal reasons (e.g., public availability would compromise patient privacy), please explain your reasons on resubmission and your exemption request will be escalated for approval.

- We have included all data underlying the findings described in the manuscript. This includes the raw and processed sequencing data, associated metadata, and analysis scripts, all of which are provided as stated in the Data and Code Availability section. As such, there is no additional data requiring deposition, and we have removed the statement, “Additional data supporting the findings of this study are available from the corresponding author upon reasonable request,” from the Data and Code Availability section.

The revised Data and Code Availability section now reads as follows (and is updated in the revised manuscript):

“The raw and processed sequencing data generated in this study have been deposited in the Gene Expression Omnibus (GEO) database under accession number GSE263649. The metadata and scripts used for snRNAseq analysis are available in the GitHub page (https://github.com/jieunesther/CC_snRNAseq/).”

**Reviewer #1:** The authors utilized snRNA-seq of a variety of different inbred mouse strains to investigate cell population differences. This is an important line of research to investigate, especially in regards to the variance observed across numerous different mouse strains. However, the author's dataset is under-analyzed with no form of gene expression analysis outside of cell type identification. Also, it is unclear how the authors could differentiate between CD13 and PECAM-1 positive nuclei at the magnification provided, with no representative images showing the differences across different strains. Thirdly, the conclusions that the authors have drawn are presumptuous and without sufficient data to support. Overall, this manuscript requires further work to aid the author's interpretations before suitable for publication.

Please see specific comments below.

Introduction
- Authors should mention relevant examples of recorded phenotypic differences between inbred mouse strains

We added the examples of recorded phenotypic differences between inbred mouse strains in the introduction (bolded parts are added).

In humans, functional genomics data from 1,866 brain samples revealed that several brain disorders were associated with alterations in brain cell type proportions (8). Different inbred mouse strains likewise show profound phenotypic differences in behavior and susceptibility to disease (9–13). **For instance, Gu et al identified CC strains with extreme seizure resistance and SUDEP (sudden unexpected death in epilepsy) susceptibility and genomic loci associated with seizure sensitivity using standardized seizure assays and quantitative trait loci (QTL) mapping (PMID: 32852103). Similarly, inbred strains such as C57BL/6N and 129S2/SvPasCrl display marked differences in hippocampal gamma oscillation power and sharp wave-ripple events compared to C57BL/6J (PMID: 37550182). Additionally, anatomical variation in cortical area maps between C57BL/6J and DBA/2J highlights the influence of genetic background on brain structure (PMID: 15774010).** While these phenotypic differences could, in part, relate to differences in brain cell composition, the extent to which strain background influences the types and proportions of cell types in the mouse brain has not been studied.

Results
- Fig.4: Authors should show representative images for 4 strains being compared in figure.

We added representative images from the four strains to Fig. 4.

- From the images shown in Fig.4, it is unclear how authors could differentiate between pericyte and endothelial nuclei due to the overlap of CD13 and PECAM-1 staining. Clearer images should be provided in supplement to support their analysis.

- We have now included representative images for each of the four strains analyzed to support our quantification of pericytes and endothelial cells. To clarify how pericytes and endothelial nuclei were distinguished, we highlight that pericytes were identified based on the colocalization of CD13 and DAPI signals, while endothelial cells were identified by the overlap of PECAM-1 and DAPI. These representative images have been added to the main figure panel (Figure 4B and 4C as shown).

- The authors should analyze gene expression changes across strains to identify potential pathways that have been dysregulated by differing population numbers.

- We focused our differential gene expression (DEG) analysis on pericytes and endothelial cells by comparing strains with high versus low proportions of each cell type. This targeted approach was taken due to the large number of potential comparisons, which are beyond the scope of a study specifically focused on vascular cell types. However, we have made all relevant data publicly available, so that other researchers can perform additional comparisons between strains of interest to explore broader differences in cell type proportion or transcriptional profiles.

To investigate whether specific pathways are dysregulated in strains with varying pericyte or endothelial cell proportions, we grouped CC009 and CC025 as high-pericyte/high-endothelial strains, and CC045 and CC046 as low-pericyte/low-endothelial strains. Given the relatively small representation of these cell types in our dataset—pericytes comprising 0.165% and endothelial cells 1.62%—the number of cells available for differential gene expression (DEG) analysis per strain was limited.

Despite this limitation, we performed DEG analysis between the high and low groups using a stringent threshold (|log₂FC| > 1.5, adjusted p-value < 0.05). This yielded one DEG in pericytes (Hexb) and 30 DEGs in endothelial cells. When visualized, several endothelial cell DEGs were specifically enriched in the low-endothelial strains (CC045 and CC046). Among these, Hexb is of particular interest: previous work has shown that endothelial-specific expression of Hexb can rescue neurological phenotypes in Sandhoff disease mouse models (Dogbevia et al., 2019), underscoring a critical role for Hexb in neurovascular health. Its upregulation in low-endothelial strains may reflect a compensatory response to impaired endothelial function.

Additionally, we observed elevated expression of Il31ra in the low-endothelial group. Given its involvement in inflammatory signaling, this may suggest enhanced immune–vascular crosstalk or impaired barrier integrity in these strains—potential contributors to altered neural homeostasis. These findings point toward transcriptional adaptations that may arise in response to strain-specific deficits in vascular cell populations.

To highlight transcriptional features enriched in strains with lower pericyte/endothelial cell proportions, we included representative DEGs from the low group in the dot plot shown in Figure 3D.

- Supplemental Fig.4: Differences in neuronal subtype population sizes are identified but authors should confirm by FISH or IHC, as they have attempted to do with the pericytes and endothelial cells.

- This analysis is beyond the scope of the current study. We prioritized validating the two most prominent cell composition differences observed between strains—pericytes and endothelial cells—through orthogonal methods. While neuronal subtype differences were also detected, they were of smaller magnitude. Further investigation of these differences can be examined in future studies.

- Overly speculative without authors providing substantial evidence from their results. For example, authors identify neurovascular issues are associated with Alzheimer’s disease using Yang et al., 2022 and that their dataset expressed pericyte markers more closely aligned with T-pericytes. Yet, the authors did not present gene expression data outside of cell-type identification.

- As noted in the Discussion, “pericytes expressed several T-pericyte markers (Slc6a1, Slc1a3), suggesting that pericytes in our snRNA-seq mouse brain dataset more closely resemble human T-pericytes than M-pericytes, consistent with findings in the literature (Yang et al., 2022).” However, we did not include supporting gene expression data in the original submission. To address this concern, we have now added a supplementary figure that shows the expression of representative T-pericyte markers (e.g., Slc6a1, Slc1a3, Slc20a2, Slc12a7, Slc6a13) and M-pericyte markers (Col4a1, Col4a2, Col4a3, Col4a4, Lama4, Adamts1) in our snRNAseq pericyte cluster (Supplementary Fig 6), showing stronger expression of some T-pericyte markers than M-pericyte markers. This addition strengthens our interpretation and provides the necessary evidence for the statement.

**Reviewer #2:** The authors surveyed the cortical cellular composition of 14 Collaborative Cross mice and further validated the differences in pericyte and endothelial cells using IHC. This information is critical to understand the potential cellular mechanism behind phenotype diversity found in this population of mice. Some outstanding questions remain to be addressed:

What is the selection criteria of the 14 strains of CC. Are there particular neural trait of interest that the authors are looking into within this population of mice?

- As we noted in the introduction, most single cell sequencing studies made use of one mouse strain (C57Bl/6). The extent to which brain cell composition differs as a function of strain background has never been explored before. The CC mice are housed at UNC. Periodically, UNC issues a call for pilot proposals to make use of the CC mice for new studies. We thus selected these 14 CC strains because all were white-coated (relevant to a different study we sought to perform) and were available for distribution as part of this pilot program. We thus initiated this study to address the primary question of how strain variation impacts brain cell composition, and to determine if genetic variation acts to a greater extent on certain brain cell types. Behavioral studies with these strains are well-beyond our goal. Our goal for this study was to determine if genetic variation among CC strains can contribute to measurable differences in brain cell type proportions. We are now the first to show that cells of the neurovascular unit are particularly sensitive to strain background.

Why the excitatory and inhibitory neurons being the most conserved cell population across strains? Is it because they comprise the large majority of the cells and tolerant to any small changes if there's any?

- Excitatory and inhibitory neurons make up the largest proportion of cells in our snRNA-seq dataset, which may contribute to their relative stability across strains. However, despite the overall conservation, we did identify specific neuronal subtypes and clusters that exhibited statistically significant differences in abundance across strains. These findings are described in the Results and further support the notion that even within broadly conserved cell types, meaningful inter-strain variation exists.

Lack of control/reference strain. The authors mentioned the commonly used laboratory inbred mice in the neuroscience studies like B6J and 129. So it is important to know where do reference strains like B6J and 129 fall in the cell composition spectrum of principal/neuronal subtypes among the phenotyped CC? Can they take advantage of existing single nucleus seq data of B6J and compare to other CC strains?

- As our primary goal was to compare across CC mouse strains, adding additional strains was deemed scientifically unnecessary. Our goal was not to compare CC to commonly used strains, and doing so would not change our conclusions with this diverse selection of CC strains. CC mice were derived from eight founder inbred strains, including C57BL/6J and 129S1/SvImJ. Use of CC mice thus allows investigators to study the range of phenotypes that originate from the eight founder strains.

While comparing CC strains to publicly available snRNA-seq datasets from these reference strains would be informative, differences in brain dissection protocols, anatomical regions profiled, and batch effects make direct comparisons challenging and potentially misleading. To mitigate these technical issues, we analyzed all 14 CC strains under the same experimental conditions, including standardized cortical dissections and sequencing pipelines. We also normalized each strain’s cell type composition to the mean across all 14 strains, allowing us to identify consistent and strain-specific deviations.

Have any neuronal/behavioral phenotypes that have been identified in any of the CC009, CC025, CC046, CC045 that can be explained by the differential cellular composition?

- Correlating the observed cellular composition differences with known neuronal or behavioral phenotypes is beyond the current scope of our study. That said, we agree that integrating transcriptomic and phenotypic data across CC strains is a promising direction for future research and could yield insights into genotype-phenotype relationships in the brain. Indeed, we are colleagues with the PI who maintains the CC mice and who devised the CC project. We suggested that he maintain a database of physiological, behavioral, and other phenotypes so that people can do exactly what you suggest—draw correlations between gene expression, cell composition and phenotypic traits of interest. This database does not exist, so we cannot perform the correlations you asked about at the present time.

Please cite and discuss other brain/vascular related phenotypic diversity that have been published in CC or their founder strains that pertain to the scope of this study: PMID: 34321020, PMID: 36275853, and PMID: 38185315.

- We thank the reviewer for pointing us to these relevant studies. As suggested, we have cited and incorporated discussion of these publications into the revised manuscript to highlight previously reported brain and vascular phenotypic diversity in CC strains and their founders. These studies further underscore the utility of the CC population in exploring genetically driven variation in brain structure and function.
